# Effect of Emerging Contaminants (Sucralose) at Relevant Concentrations on Functional Properties in Fish Muscle of Common Carp (*Cyprinus carpio*)

**DOI:** 10.3390/foods14193387

**Published:** 2025-09-30

**Authors:** Karinne Saucedo-Vence, Octavio Dublán-García, Ana Gabriela Morachis-Valdez, Daniel Díaz-Bandera, Francisco Antonio López-Medina, Guadalupe López-García, Andrea Yazmín Guadarrama-Lezama, Gerardo Heredia-García, Angel Santillán-Álvarez, Leobardo Manuel Gómez-Oliván, Elvia Alba-Rojas

**Affiliations:** 1 Laboratorio de Alimentos y Toxicología Ambiental, Facultad de Química, Universidad Autónoma del Estado de México, Carr. Toluca-Ixtlahuaca Km. 15, El Cerrillo Piedras Blancas, Toluca 50200, Mexico; karinne.saucedo@utvtol.edu.mx (K.S.-V.); ddiazb@uaemex.mx (D.D.-B.); flopezmedina92@gmail.com (F.A.L.-M.); logg_sf@hotmail.com (G.L.-G.); ayguadarramal@uaemex.mx (A.Y.G.-L.); gherediag516@alumno.uaemex.mx (G.H.-G.); lmgomezo@uaemex.mx (L.M.G.-O.); 2 Unidad Académica de Capulhuac, Universidad Tecnológica del Valle de Toluca (UTVT), Calle s/n, 611 Oriente de Mexico, Lomas de San Juan, Capulhuac de Mirafuentes 52700, Mexico; 3 División de Gastronomía, Tecnológico Nacional de México (TecNM), Tecnológico de Estudios Superiores de Valle de Bravo, Km 30 de la Carretera Nacional Federal Monumento-Valle de Bravo, Ejido de San Antonio de La Laguna, Valle de Bravo 51200, Mexico; angel.sa@vbravo.tecnm.mx; 4 Conjunto SEDAGRO, Edificio Central S/N Ex. Rancho Manzana 001, San Lorenzo Coacalco, Coacalco 52140, Mexico; earojas.sma@edomex.gob.mx

**Keywords:** sucralose, emerging contaminants, oxidative stress, physicochemical properties, protein oxidation

## Abstract

Sucralose, a persistent and widely used artificial sweetener, has emerged as a significant contaminant in aquatic environments, raising concerns about its ecological and physiological effects on aquatic species. This study investigates the impact of environmentally relevant concentrations of sucralose on the muscle quality of common carp (*Cyprinus carpio*), a bioindicator species. Using High-Performance Liquid Chromatography (HPLC), sucralose was quantified in water and fish muscle tissues, revealing its persistence and bioaccumulation. Sucralose exposure disrupted critical physicochemical, textural, and structural properties of fish muscle. Protein carbonyl content increased up to 10-fold, while lipid peroxidation levels rose significantly, indicating oxidative stress. Sulfhydryl groups were reduced by more than 40%, and water-holding capacity decreased by 12%, compromising muscle functionality. Textural profile analysis revealed alterations in hardness, cohesiveness, and elasticity, linked to covalent bond formation induced by protein oxidation. Furthermore, electrophoretic analysis confirmed myosin degradation, underscoring sucralose’s role as a pro-oxidant, even at low concentrations. These findings demonstrate that sucralose can adversely affect aquatic organisms by impairing muscle integrity, with potential consequences for their survival, ecological roles, and food web dynamics. This study underscores the urgent need to regulate and monitor artificial sweeteners in aquatic systems to mitigate long-term ecological impacts.

## 1. Introduction

Artificial sweeteners are synthetic compounds widely used in food, beverages, pharmaceuticals, and personal care products due to their high-intensity sweetness and low caloric content. Among the most prevalent are sucralose (SUC), aspartame (ASP), acesulfame-K (Ace-K), and saccharin (SAC), which are approved for use in numerous countries [1,2]. Sucralose, in particular, is approximately 600 times sweeter than sucrose and is found in over 4500 food and beverage products worldwide, often combined with other sweeteners [3,4]. Its chemical stability and resistance to heat and pH variations make it highly suitable for diverse applications [5]. However, these same properties contribute to its persistence in the environment, raising concerns about its classification as an emerging contaminant [6].

Emerging contaminants, such as sucralose, are unregulated pollutants that enter ecosystems in significant amounts due to anthropogenic activity [4,7]. Sucralose has been detected in wastewater treatment effluents (1 µg/L DE; up to 46,100 ng/L USA; 10,800 ng/L SE; 21,000 ng/L CAN and 18,000 ng/L CN) even groundwater, with reported concentrations ranging from 0.05 to 155 µg/L in various regions [8,9,10]. These levels highlight its resistance to degradation during water treatment processes and its potential for long-term accumulation in aquatic environments. Unlike other contaminants, sucralose does not break down easily, posing a unique challenge to ecosystem health and environmental sustainability [4,6].

Studies have shown that persistent contaminants can induce oxidative stress, alter metabolic pathways, and compromise protein functionality in aquatic species [11,12]. Myofibrillar proteins, such as myosin, are particularly susceptible to oxidative damage, which can impair their structural and functional properties [13,14]. This has profound implications for the health and survival of aquatic species, as well as for the trophic dynamics of ecosystems where these organisms play a critical role [11]. Therefore, the presence of substances as sucralose, highly persistent in the environment can induce changes in protein structure in organisms like *Cyprinus carpio*, used as a bioindicator because of its wide distribution in aquatic systems, easy availability, high economic value, and growth traits, and its application in toxicity studies [15,16,17,18].

This study focuses on the impact of sucralose exposure at environmentally relevant concentrations on the muscle quality of common carp (*Cyprinus carpio*). Specifically, the research evaluates structural changes in myofibrillar proteins, including protein carbonyl content (PCC), lipid peroxidation (LPX), sulfhydryl (SH) group content, water-holding capacity (WHC), and textural properties. By linking these biochemical and physicochemical changes to the persistence of sucralose in aquatic environments, this study provides critical insights into the food characteristics exposed to artificial sweeteners and emphasizes the urgent need for their regulation and monitoring.

## 2. Materials and Methods

### 2.1. Assayed Substance

Analytical grade sucralose (C_12_H_19_Cl_3_O_8_, CAS No. 56038-13-2), molecular weight 397.63, purity ≥ 98%, was purchased from Sigma-Aldrich (St. Louis, MO, USA), as were all other reagents unless otherwise stated.

### 2.2. Species Acquirement and Maintenance

One hundred fish (*Cyprinus carpio*) were transported from aquaculture center in Tiacaque (State of Mexico) to laboratory in oxygenated water in sealed polyethylene bags, longing and weighing 19.45 ± 0.53 cm 56.82 ± 8.3 g, respectively, where they were placed in a tank (1100 L) containing dechlorinated tap water: pH 7.5–8.0, temperature 20 ± 2 °C, total hardness 18.7 ± 0.6 mg/L, alkalinity 17.8 ± 7.3 mg/L, (previously reconstituted with salts) during 45 days previous to the assay. Fish were fed Pedregal SilverTM fish food, México, MX and water was replaced daily (three fourths of the tank), following day/night photoperiod.

### 2.3. Experimental Design

For the experimental phase, three SUC exposure conditions were established and mounted in independent systems with five repetitions each. The exposure systems included T05-SUC: 0.05 µg/L and T155-SUC: 155 µg/L, in addition to the control system that was kept free of SUC. The systems consisted of 60 × 40 × 20 cm glass containers, each containing 10 juvenile stage fish (size and weight described above), a total of 300 fish available for all analyses (30 per treatment). All systems were maintained at room temperature with a natural light/dark photoperiod of 12 h:12 h and provided constant aeration, at an oxygen concentration between 80% and 90%, during the experiment no food was provided to the fish. As recommended by [19] and with approval of Colegio Estatal de Médicos Veterinarios Zootecnistas Estado de México A.C. Ethical Committee. The time of exposure to SUC was also considered in the study. For this purpose, five test organisms from each exposure system were removed at 12, 24, 48, 72, and 96 h and the response variables were evaluated.

### 2.4. Quantification of Sucralose in Water Systems and Muscle Tissue Using HPLC

The concentrations of sucralose (SUC) in the water system and muscle tissue of common carp (*Cyprinus carpio*) were determined using High-Performance Liquid Chromatography (HPLC), according to [15]. Water samples were collected from the exposure tanks at 12, 24, 48, 72, and 96 h for both exposure concentrations (0.05 µg/L and 155 µg/L), as well as from the control group. Muscle tissue samples were extracted from the dorsal region of the carp immediately after euthanasia at the same time intervals. Water samples (50 mL) were filtered through 0.45 µm nylon filters to remove particulate matter and stored at 4 °C until analysis. Muscle tissue (approximately 2 g) was homogenized in 5 mL of phosphate-buffered saline (PBS, pH 7.4) using a tissue homogenizer, followed by centrifugation at 10,000× *g* for 15 min at 4 °C. The supernatant was collected and filtered through 0.22 µm syringe filters before HPLC analysis.

The quantification of SUC was performed using an HPLC system equipped with a UV detector. Separation was achieved on a C18 reverse-phase column (250 mm × 4.6 mm, 5 µm particle size), using a mobile phase consisting of methanol and water (70:30 *v*/*v*) at a flow rate of 1.0 mL/min. The injection volume was 20 µL, and SUC was detected at a wavelength of 227 nm, with a run time of 10 min per sample. Calibration curves were prepared using serial dilutions of a SUC stock solution (1000 µg/mL), ranging from 0.001 µg/mL to 200 µg/mL, and demonstrated excellent linearity (R^2^ > 0.99). SUC concentrations in water and muscle samples were quantified based on the peak area and compared against the standard curve.

Quality control procedures included analyzing spiked water and muscle samples with known SUC concentrations, yielding recovery rates between 92% and 98%, confirming the reliability of the method. All analyses were performed in triplicate to ensure reproducibility and minimize variability.

### 2.5. Common Carp Muscle Extraction

The fish were anesthetized in a container with water and 0.01% eugenol and were sacrificed, muscle was extracted without scales or bones with scalpel, 10% of the muscle was placed in a phosphate buffer solution (pH 7.4), they were homogenized and then they were centrifuged at 12,500 rpm and −4 °C for 15 min. The centrifuged samples were kept frozen, and the supernatant was used to evaluate the following biomarkers of oxidative stress: hydroperoxide content (HPC), thiobarbituric acid assay (TBARS), carbonylated protein content (CCP). The remaining muscle was pooled and stored at −70 °C for later evaluation of physicochemical and textural properties.

### 2.6. PCC Determination

PCC was determined by the method described by [20], modified by [21,22], 150 μL (10 mM DNPH prepared in 2M HCl) were added to 100 μL of the supernatant; resting at room temperature for 1 h in light absent to allow the derivatization of carbonyl groups, 500 μL of trichloroacetic acid (20%) were added, and solution rested in refrigeration for 15 min. Product was centrifuged in an Eppendorf 5810 R centrifuge (15-amp version, Hamburg, Germany) at 1100× *g* for 5 min. With ethanol/ethyl acetate (1:1) was used to wash the bud to remove unreacted DNPH and lipophilic impurities until the supernatant completely colorless, then, dissolved in 1 mL guanidine solution (6 M, pH 2.3) and incubated at 37 °C for 30 min. Readings at 366 nm were made and results were obtained using molar extinction coefficient (MEC) of 21,000 M/cm as μM of reactive carbonyls formed (C=O)/mg protein.

### 2.7. LPX Determination

The method described by [23], was used. To 100 µL of uncentrifuged sample, 150 mM Tris-HCl buffer at pH 7.4 was added until a 1-mL volume was attained. Next, 2 mL of TBA-TCA reagent [0.375% thiobarbituric acid (TBA) in 15% TCA] was added, and the mixture was heated in boiling water and then incubated in a water bath at 37 °C for 30 min. The sample was centrifuged at 3500 rpm for 10 min, and absorbance was read at 535 nm. Results were expressed as mM malondialdehyde (MDA)/mg protein, using the molar extinction coefficient (MEC) of 1.56 × 10^5^/M/cm.

### 2.8. Total SH Content

According to [24], myofibrillar protein solution (5 mg/mL) was reacted at ambient temperature for 30 min with 9 mL Tris-glycine buffer at pH 8.0 (6.9 g glycine, 1.2 g EDTA/L, 480 g urea, and 10.4 g Tris-HCl). A total of 0.05 mL Ellman’s reagent (4 mg DTNB/mL) was added to 3-mL aliquots and reacted in the dark for 30 min. The reaction was read at 412 nm using a TU-1800 spectrophotometer (Beijing Purkinje General Instrument, Beijing, China). Total SH content was expressed as μM SH/mg total protein.

### 2.9. Total Protein Content Determination

Total protein content was determined by the biuret method, as described by [25], Dilutions (1:15 and 1:20) from 1 g of protein were made with NaOH (10%) and NaCl (0.6 M) solutions. The mixture was reacted for 30 min and measured at 540 nm using a GENESYSTM 10S Vis spectrophotometer (Thermo Scientific, Madison, WN, USA). Protein concentration was determined using a BSA standard curve.

### 2.10. pH

A Conductronic pH 120 potentiometer was used to determine pH. Carp muscle (10 g) was mixed with 90 mL distilled water for 1 min. The mixture was filtered to remove connective tissue, as described by [25], pH was measured in triplicate.

### 2.11. Solubility

Solubility was determined using the method proposed by [26], in which MP samples of 5 mg/mL at a previously determined pH of 7 centrifuged at 3500 rpm/15 min/4 °C to prevent denaturation. Biuret method was used to quantify supernatant protein content in the supernatant expressing solubility as the ratio between the supernatant protein content in and the uncentrifuged protein content in the sample x 100.

### 2.12. WHC

Water Holding Capacity (WHC) was measured following [27]; 20 g of fish muscle was minced, and 5 g of the minced sample was placed in separate centrifuge tubes and mixed with 8 mL of 0.6 M NaCl solution in a bath with ice, undergoing 1 min of stirring with a glass rod. The mixture was allowed to settle during 30 min, stirred again for 1 min, and then centrifuged at 4 °C for 3500 rpm. Results were expressed as NaCl volume retained per 100 g of sample after decanting.

### 2.13. MP Extraction

MP was extracted using methodology cited in [28], with certain modifications. Using 100 mL (25 mM at pH 7) of phosphate buffer solution and 0.9% NaCl (temperature 4 °C or less), common carp muscle (100 g) was homogenized plus 200 mL distilled water, using a homogenizer in order to incorporate muscle completely into the buffer. Using a 1-L beaker, the resulting extract was stirred constantly for 15 min into an ice bath. Connective tissue was eliminated by filtering the homogenate, then centrifuged at 15 min/4 °C/3500 rpm. Protein concentration was quantified in the resulting precipitate by the biuret method.

### 2.14. Gelation of Proteins

After protein extraction, the extracts were poured into glass containers with screw lids and heated gradually in a double boiler until core temperature reached 80 °C to induce gelation. Containers were removed and placed in an ice bath, then stored at 4 °C or less until texture analysis, in order to mature gel structure as described by [29].

### 2.15. TPA

MP samples were transferred into glass vials (30 mm diameter × 35 mm height). The gels formed at 80 °C in the course of a 30-min water bath. After heating, the gels were chilled and left at rest during the night at 4 °C, samples were prepared forming 2.5-cm long cylindrical temperate (20–25 °C) forms prior TPA analysis, using a TA-XT2 v2.63 texture analyzer (Texture Technologies, Scarsdale, NY, USA) equipped with a 25 N load cell. Two compressions (1 mm/s) were made with a 1.25 cm spherical piston, with a 5-s wait between each other, as described by [30]. Data obtained were scanned with v1.20 (Stable Micro Systems, Surrey, UK) to calculate texture attributes.

### 2.16. Electrophoresis

Samples of the gels obtained, and the supernatants were analyzed by SDS-PAGE (sodium dodecyl sulfate-polyacrylamide gel electrophoresis), using the procedure in, to analyze 15-µL volumes of sample and molecular weight markers [31]. The analysis was performed using a Mini-PROTEAN^®^ Tetra Cell system (Bio-Rad Laboratories, Inc., Hercules, CA, USA) for 40 min, 200 mV. After each run, the gels were 1 h stained using Coomassie Brilliant Blue R-250 Sigma-Aldrich, St. Louis MO, USA, at 0.1%, and then placed in 15% acetic acid-40% methanol until complete faded for clear band visualization.

### 2.17. Statistical Analysis

The effect of exposure time and SUC concentration on the Response Variables were statistically evaluated through a multifactorial design using two-way analysis of variance (ANOVA). For the comparison between treatments (Concentration, Time, and interaction) the Bonferroni test was used considering differences for values with *p* < 0.05. Statistical determinations were performed using Minitab 18.1 software (USA, 2017). The data presented in the manuscript correspond to the mean values obtained from five independent replicates, which minimizes experimental variability and strengthens the validity of the conclusion.

The results were statistically evaluated through a multifactorial design to study the effect of time and concentration on the response variables with *p* < 0.05. Statistical analyses were conducted using Minitab 18.1 (USA, 2017).

## 3. Results

The concentrations of sucralose (SUC) in the water system and muscle tissue of common carp (*Cyprinus carpio*) were determined using HPLC. The results are summarized in Table 1, which demonstrates the persistence of SUC in the aquatic environment and its bioaccumulation in fish tissue over the exposure period (12–96 h).

In the control group, SUC was not detected in either the water system or muscle tissue, confirming the absence of background contamination. At the lower exposure concentration (0.05 µg/L), SUC levels in the water system remained stable throughout the exposure period, ranging between 0.02 and 0.04 µg/L. Correspondingly, low but detectable levels of SUC were observed in the muscle tissue, starting at 12 h (0.0010 ± 0.0001 µg/g) and persisting with minor fluctuations up to 96 h. These results indicate that even low environmental concentrations of SUC can penetrate biological tissues, demonstrating its bioavailability and potential for bioaccumulation.

At the higher exposure concentration (155 µg/L), SUC levels in the water system were consistently high, ranging from 98.3 ± 2.9 µg/L to 132.2 ± 3.1 µg/L. In muscle tissue, SUC concentrations increased significantly over time, reaching a maximum of 7.6 ± 1.2 µg/g at 48 h and stabilizing slightly at 72 and 96 h (8.3 ± 2.3 µg/g and 8.1 ± 1.5 µg/g, respectively). This trend highlights the capacity of fish muscle to accumulate SUC when exposed to elevated environmental concentrations, potentially exceeding the organism ability to metabolize or excrete the compound efficiently.

The comparison between the two exposure concentrations reflects the concentration-dependent behavior of SUC in aquatic systems. While the lower concentration resulted in minimal accumulation, the higher concentration demonstrated a marked ability to persist in water and accumulate in muscle tissue. These findings confirm the environmental persistence of SUC and its bioaccumulative potential in aquatic organisms, even at relatively low exposure levels.

The two-way ANOVA revealed that both sucralose concentration and exposure time significantly affected all measured variables (*p* < 0.05). Significant interaction effects (concentration × time) were also observed for PCC, LPX, SH content, WHC, and texture parameters, indicating that the influence of sucralose on muscle quality depends on both concentration and exposure duration.

**Figure 1:**
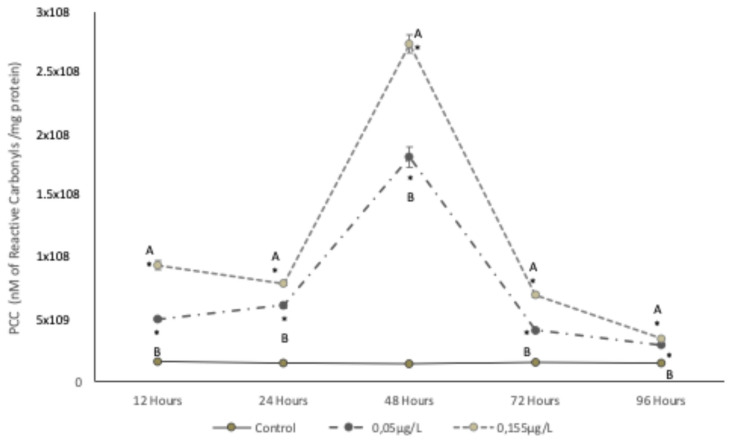
Changes in protein carbonyl content (PCC) of *Cyprinus carpio* muscle, expressed in nM of reactive Carbonyls/mg protein, upon exposure to two concentrations of SUC (0.05 µg/L and 155 µg/L) for 12, 24, 48, 72 and 96 h. Data are presented as mean ± SD (n = 5). Different letters indicate significant differences among treatments (*p* < 0.05). Asterisks indicate significant differences compared with the control group (*p* < 0.05).

The PCC values in carp muscle are shown in Figure 1. At a concentration of 0.05 µg/L SUC (the lowest concentration found in water reservoirs), significant increases of 241.3%, 294.3%, 1149.2%, 160.9%, and 110.9% (*p* < 0.05) were observed from 12 to 96 h compared to the control. At 155 µg/L SUC (the highest concentration found in water reservoirs), significant increases of 296.01%, 111.3%, 559.5%, and 220.7% were observed from 12 to 72 h, followed by a decrease of 68.7% at 96 h (*p* < 0.05).

**Figure 2:**
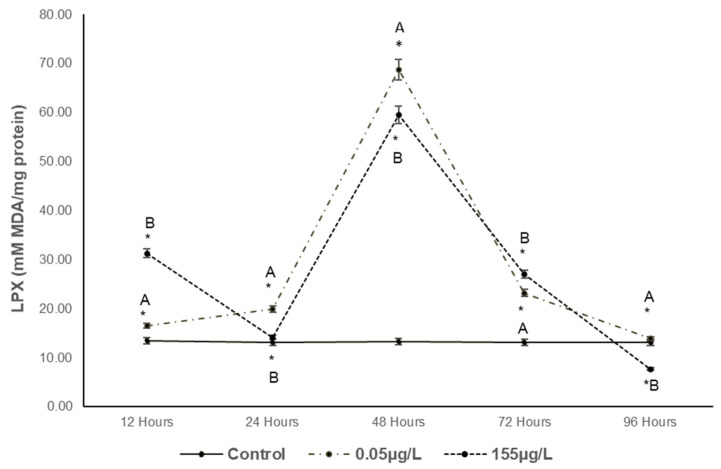
Changes in lipid peroxidation (LPX) of *Cyprinus carpio* muscle expressed as nM MDA/mg protein (MDA = malondialdehyde) exposed to two concentrations of SUC (0.05 µg/L and 155 µg/L) for 12, 24, 48, 72 and 96 h Data are presented as mean ± SD (n = 5). Different letters indicate significant differences among treatments (*p* < 0.05). Asterisks indicate significant differences compared with the control group (*p* < 0.05).

LPX results (Figure 2) show significant increases of 123.8%, 151.2%, 517.1%, 176.01%, and 106.01% (*p* < 0.05) compared to the control at the lowest concentration (0.05 µg/L) from 12 to 96 h. At the highest concentration (155 µg/L), significant increases of 233.5%, 107.7%, 447.9%, and 372.2% (*p* < 0.05) were observed from 12 to 72 h, followed by a decrease of 41.7% at 96 h (*p* < 0.05).

**Figure 3:**
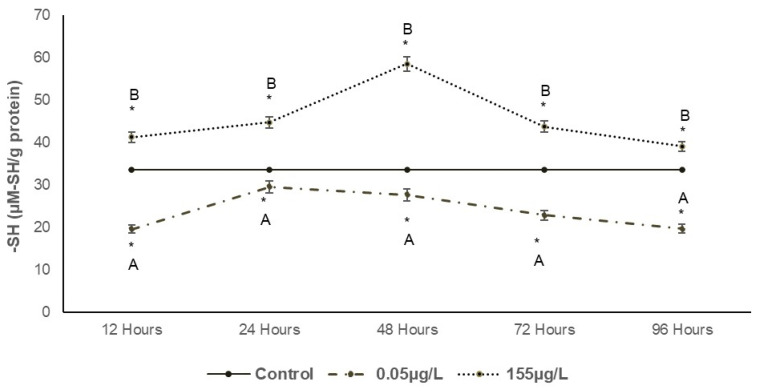
Changes in sulfhydryl group (-SH) in *Cyprinus carpio* muscle expressed as µM-SH/mg protein exposed to two different concentrations of SUC (0.05 µg/L and 155 µg/L) for 12, 24, 48, 72 and 96 h. Data are presented as mean ± SD (n = 5). Different letters indicate significant differences among treatments (*p* < 0.05). Asterisks indicate significant differences compared with the control group (*p* < 0.05).

Figure 3 illustrates the total SH content in carp muscle. At the lowest concentration (0.05 µg/L SUC), significant decreases of 41.83%, 12.12%, 5.62%, 32.06%, and 29.58% (*p* < 0.05) were observed compared to the control during the exposure period. In contrast, the 155 µg/L concentration induced significant increases of 22.64%, 33.06%, 74.11%, 30.15%, and 16.19% (*p* < 0.05) at the corresponding exposure times.

**Figure 4:**
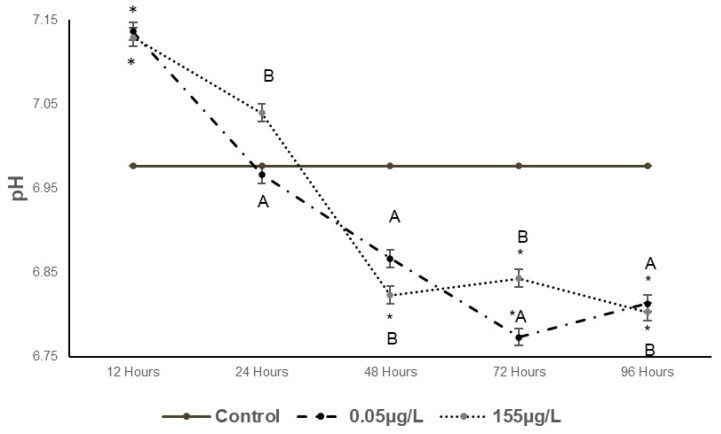
Changes in pH in *Cyprinus carpio* muscle exposed to two different concentrations of SUC (0.05 µg/L and 155 µg/L) for 12, 24, 48, 72 and 96 h. Data are presented as mean ± SD (n = 5). Different letters indicate significant differences among treatments (*p* < 0.05). Asterisks indicate significant differences compared with the control group (*p* < 0.05).

The pH behavior across the five exposure times is shown in Figure 4. At the lowest concentration (0.05 µg/L SUC), a significant increase of 2.29% (*p* < 0.05) compared to the control was observed at 12 h, followed by significant decreases of 0.14%, 1.57%, 2.91%, and 2.34% from 24 to 96 h. At the highest concentration (155 µg/L SUC), increases of 2.19% and 0.90% were recorded at 12 and 24 h, respectively, while decreases of 2.19%, 1.91%, and 2.48% were observed from 48 to 96 h.

**Figure 5:**
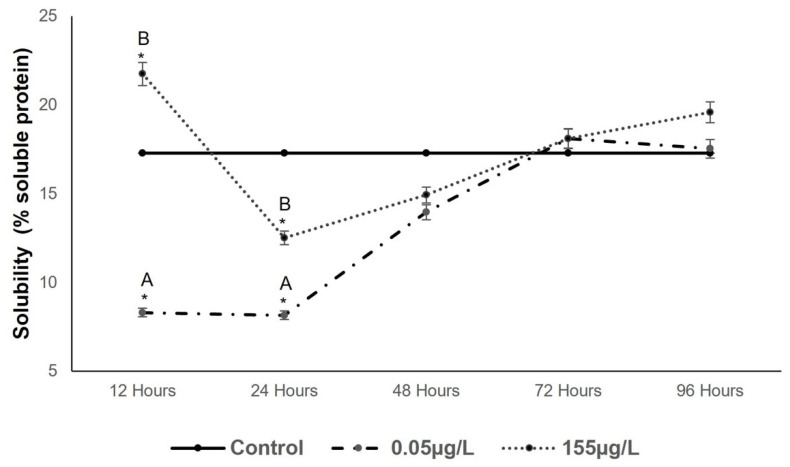
Changes in Solubility in *Cyprinus carpio* muscle expressed as % of soluble protein to two different concentrations of SUC (0.05 µg/L and 155 µg/L) for 12, 24, 48, 72 and 96 h. Data are presented as mean ± SD (n = 5). Different letters indicate significant differences among treatments (*p* < 0.05). Asterisks indicate significant differences compared with the control group (*p* < 0.05).

Figure 5 illustrates the solubility behavior of carp muscle proteins. At the 0.05 µg/L concentration, significant decreases of 51.97%, 52.83%, and 19.30% (*p* < 0.05) relative to the control were observed from 12 to 48 h, while increases of 4.74% and 1.38% were recorded at 72 and 96 h, respectively. At the highest concentration (155 µg/L SUC), significant increases of 25.75%, 4.63%, and 13.23% were observed at 12, 72, and 96 h, respectively, whereas decreases of 27.65% and 13.63% occurred between 24 and 48 h.

**Figure 6:**
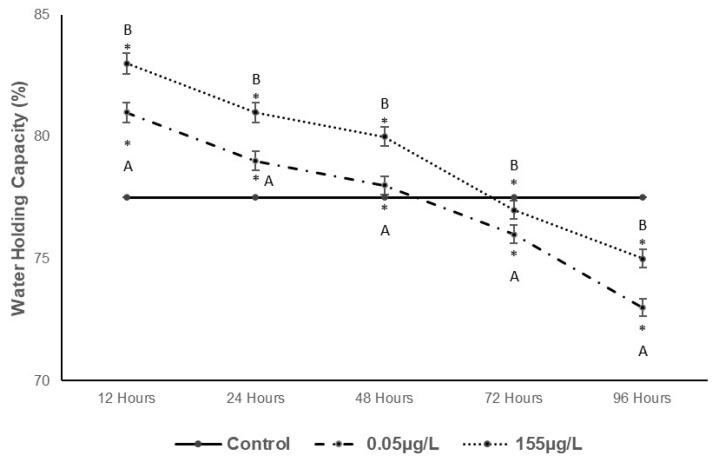
Changes in WHC in *Cyprinus carpio* muscle expressed as NaCl/100 g retention exposed to two different concentrations of SUC (0.05 µg/L and 155 µg/L) for 12, 24, 48, 72 and 96 h. Data are presented as mean ± SD (n = 5). Different letters indicate significant differences among treatments (*p* < 0.05). Asterisks indicate significant differences compared with the control group (*p* < 0.05).

Figure 6 shows the water-holding capacity (WHC) in common carp muscle. At the lowest concentration (0.05 µg/L SUC), significant increases of 4.51%, 1.21%, and 0.62% (*p* < 0.05) compared to the control were observed from 12 to 48 h, followed by decreases of 1.95% and 5.82% from 72 to 96 h. At the highest concentration (155 µg/L SUC), increases of 7.07%, 4.49%, and 3.20% were observed from 12 to 48 h, with decreases of 0.66% and 3.24% recorded from 72 to 96 h.

Table 2 presents the behavior of texture profile analysis (TPA). At the 0.05 µg/L concentration, significant decreases in hardness of 5.73%, 7.37%, 9.01%, and 2.45% (*p* < 0.05) were observed from 12 to 72 h, followed by an increase of 9.01% at 96 h. At the highest concentration (155 µg/L), increases in hardness of 18.03% and 22.13% were recorded from 72 to 96 h, while decreases of 2.45% and 9.83% were observed from 24 to 48 h. Cohesiveness significantly increased across all exposure times at both concentrations. For the 0.05 µg/L concentration, increases of 200%, 190.90%, 181.81%, 218.18%, and 236.36% were observed from 12 to 96 h. Similarly, at the 155 µg/L concentration, increases of 209.09%, 227%, 236.36%, 236.36%, and 227% were recorded during the same time intervals. Elasticity showed significant decreases of 8.69%, 10.8%, and 15.21% from 12 to 48 h at the 0.05 µg/L concentration, followed by increases of 2.17% and 10.86% from 72 to 96 h. At the 155 µg/L concentration, elasticity increased by 6.52% at 96 h, while decreases of 4.34%, 8.69%, and 13.04% were observed from 12 to 48 h, with no variation at 72 h. Chewiness exhibited significant increases at all exposure times, ranging from 166% to 316% at the 0.05 µg/L concentration, and from 200% to 316% at the 155 µg/L concentration. Finally, gumminess also increased significantly (*p* < 0.05) throughout the exposure period. At the 0.05 µg/L concentration, increases of 115.38%, 176.92%, 169.23%, 223.07%, and 276.92% were observed from 12 to 96 h. Similarly, at the 155 µg/L concentration, gumminess increased by 223.07%, 230.76%, 207.69%, 323.07%, and 300% during the same time intervals.

## 4. Discussion

### 4.1. Persistence and Bioaccumulation of Sucralose in Water Systems and Muscle Tissue of Common Carp Detected by HPLC

The concentrations of sucralose (SUC) detected in the water system and the muscle tissue of common carp (*Cyprinus carpio*) at different exposure times (12–96 h) are shown in Table 1. In the control group, no detectable levels of SUC were observed in either the water system or muscle tissue, confirming the absence of background contamination.

At the lower concentration (0.05 µg/L), SUC levels in the water system remained stable over time, with values ranging between 0.02 and 0.04 µg/L. Correspondingly, low but detectable levels of SUC were observed in the muscle tissue as early as 12 h (0.0010 ± 0.0001 µg/g), with minor increases up to 96 h. These results demonstrate that even low environmental concentrations of SUC can penetrate biological matrices, indicating its bioavailability and potential for bioaccumulation.

At the higher concentration (155 µg/L), SUC levels in the water system remained consistently high, ranging between 98.3 ± 2.9 µg/L and 132.2 ± 3.1 µg/L throughout the exposure period. In muscle tissue, SUC concentrations increased significantly over time, with the highest levels observed at 48 h (7.6 ± 1.2 µg/g) before stabilizing slightly at 72 and 96 h. This trend highlights the capacity of fish muscle to accumulate SUC when exposed to elevated environmental concentrations.

The differences between the two exposure concentrations reflect the concentration-dependent behavior of SUC in aquatic systems and its potential for accumulation in biotic tissues. These findings confirm the persistence of SUC in water systems and its bioaccumulation in muscle tissue, even at low environmental concentrations.

### 4.2. Physicochemical and Functional Properties

Overall, the two-way ANOVA confirmed that both sucralose concentration and exposure time significantly influenced the measured variables. Moreover, significant interaction effects (concentration × time) were observed for oxidative stress markers (PCC, LPX), SH content, WHC, and texture parameters. This indicates that the impact of sucralose on muscle quality cannot be explained solely by concentration or time independently, but rather by the combined effect of both factors.

#### 4.2.1. PCC and LPX

Exposure of carp muscle to SUC concentrations (0.05 µg/L and 155 µg/L) resulted in significant lipid and protein oxidation, with increases observed between 12 and 72 h. Similar oxidative activity has been reported by [32], in red drum fish exposed to peroxide radicals, as well as in studies involving diclofenac [33] and other emerging contaminants in carp. This pattern of oxidative damage is comparable to that induced by frozen storage in surimi [34], carp fillets [29], and processed mackerel [35].

The observed increases in PCC and LPX may be attributed to the effects of SUC on enzyme expression, particularly P-glycoprotein (P-gp) and cytochrome P450 (CYP3A, CYP2D), which have a known specificity for chlorinated hydrocarbons [36,37,38]. These changes can alter protein structural properties, modify amino acid side chains, promote the formation of new protein polymers, increase PCC levels, enhance proteolytic susceptibility, and reduce solubility.

#### 4.2.2. SH Groups

Exposure to SUC had a significant effect (*p* < 0.05) on sulfhydryl (SH) groups, as observed in Figure 3. A decrease in SH groups is likely due to protein oxidation driven by radical-mediated mechanisms, which form disulfide bridges from SH groups. Cysteine, known for its high nucleophilic property, is one of the amino acids most susceptible to oxidation by reactive oxygen species. Consequently, the myosin head becomes particularly vulnerable to this reaction [14,39].

Disulfide bonds play a critical role in the tertiary structure of proteins, enhancing their thermodynamic stability when other forces of attraction are weak and contributing to the firmness of muscle tissue. However, when an increased quantity of disulfide bonds is formed, proteases may be unable to effectively degrade proteins, leading to reduced functional properties [28,40].

#### 4.2.3. pH

A significant decrease in pH (*p* < 0.05) was observed (Figure 4), which may be attributed to the accumulation of lactic acid produced during anaerobic glycolysis in muscle tissue. Glycogen, the primary energy source stored in animal tissues, is metabolized into lactic acid during glycolysis, leading to a reduction in pH. This process can be exacerbated by stress caused by exposure to various compounds, including metals, wastewater, and pesticides [41,42,43]. Ali et al. [43], reported enzymological alterations in *Cyprinus carpio* and *Labeo rohita* cultured in oxidation ponds, highlighting changes in protein metabolism under wastewater exposure. These findings support the notion that environmental contaminants can disrupt enzymatic activity, which may indirectly contribute to oxidative stress and muscle quality deterioration. Additionally, the presence of SUC in fish has been linked to oxidative stress, which may further contribute to these metabolic changes [13,44].

#### 4.2.4. Solubility

Myosin, the primary component of fish myofibrillar proteins (MP), is critical for maintaining the functional stability of muscle and reflects the biochemical properties of proteins in this food matrix [45,46]. Lipid oxidation produces malondialdehyde (MDA), which can shift the isoelectric point of myosin toward the acidic side due to the loss of the electrostatic charge of lysine residues [47].

Figure 5 highlights significant differences (*p* < 0.05) in soluble protein content compared to the control during the first exposure period at both SUC concentrations. After 24 h, these differences were only evident at the lower concentration (0.05 µg/L), with no differences observed at later exposure times.

According to [48], reactive oxygen species induce substantial oxidative damage, including lipid oxidation, enzyme inactivation, and protein degradation. These processes promote the formation of new protein-protein bonds and alter the concentration of soluble proteins, consistent with the observations during the initial exposure times [49].

#### 4.2.5. WHC

The ability of meat to retain inherent or incorporated water under external pressure is referred to as water-holding capacity (WHC) [50]. In food technology, WHC is a critical quality parameter that significantly influences the organoleptic properties of food products during processing and storage. As shown in Figure 6, WHC decreases with increasing exposure time. According to [51], protein oxidation is a major limiting factor that reduces the ability of myofibrils to absorb water, which likely explains the observed trend in WHC.

#### 4.2.6. TPA

Texture profile analysis (TPA) of the gel network is an effective indicator of the properties of muscle myofibrillar proteins [52] confirmed that intermolecular disulfide bonds formed during oxidation reactions contribute to the formation and elasticity of the gel network. According to [53], oxidation induced by malondialdehyde (MDA), a by-product of lipid peroxidation (LPX), can alter the physicochemical structure of the protein gel network. This includes an increase in protein carbonyl content (PCC), which results in decreased sulfhydryl (SH) groups and water-holding capacity (WHC).

In the present study, no significant differences were observed in gel properties across different exposure times and SUC concentrations compared to the control (*p* < 0.05). However, an increase in gel strength was noted, likely due to covalent bond formation during the heating process used to create the gel, which may have contributed to maintaining its structural integrity [54].

#### 4.2.7. Electrophoresis

**Figure 7:**
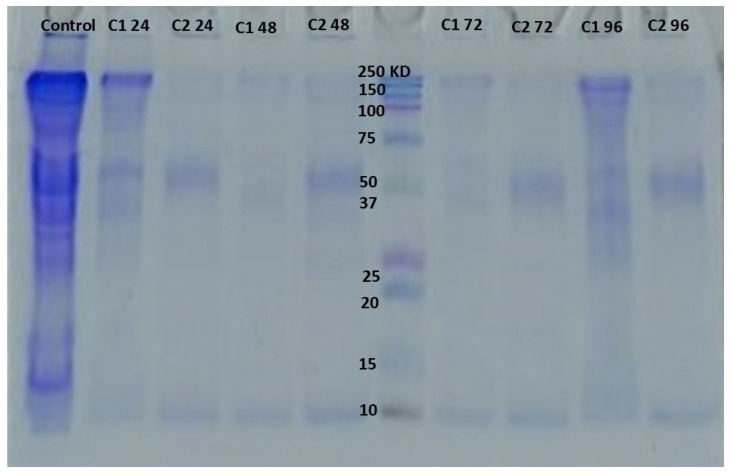
SDS-PAGE Profile of myofibrillar proteins of *Cyprinus carpio* muscle exposed to two concentrations of SUC (0.05 µg/L and 155 µg/L) for 12, 24, 48, 72 and 96 h.

SDS-PAGE is a protein separation technique based on the molecular weight of the sample. In the present study, low molecular weight bands were observed (Figure 7), indicating myosin degradation in the presence of SUC at different concentrations and exposure times. This finding suggests that SUC can increase protein carbonyl content (PCC) through the action of lipid peroxidation (LPX) by-products, as reported by [13]. These results indicate that SUC acts as a pro-oxidant, even at the lowest concentrations detected in water reservoirs. Similar findings were observed by [54].

## 5. Conclusions

This study provides robust evidence for the persistence and bioaccumulative potential of sucralose (SUC) in aquatic environments, confirming its chemical stability and limited degradation, even at environmentally relevant concentrations. Unlike other emerging contaminants that undergo partial breakdown in natural systems, SUC exhibits recalcitrant behavior, allowing it to accumulate in the muscle of *Cyprinus carpio* and reinforcing its classification as an environmentally persistent pollutant.

The bioaccumulation of SUC was not merely reflected in its detection in tissues but was also accompanied by profound biochemical and structural alterations. Elevated protein carbonyl content (PCC) and lipid peroxidation (LPX), together with the reduction of sulfhydryl groups (SH) and impaired water-holding capacity (WHC), reveal a state of oxidative stress that compromises the integrity of myofibrillar proteins. Electrophoretic analysis further confirmed the degradation of myosin, substantiating the pro-oxidant role of SUC and demonstrating its capacity to impair muscle function. These effects may negatively influence fish swimming performance, feeding capacity, and survival, ultimately undermining population stability in contaminated habitats.

From an ecological perspective, these findings are alarming. The deterioration of muscle functionality suggests that SUC exposure may reduce the adaptive capacity of aquatic species, thereby altering food web dynamics and ecosystem resilience. Moreover, its persistence and bioaccumulation highlight the risk of trophic transfer, raising concerns about potential impacts on higher-order predators, including species of commercial relevance and, eventually, human consumers.

On the regulatory level, this study underscores the urgent need to establish specific monitoring and mitigation strategies for artificial sweeteners. The inefficiency of conventional wastewater treatment plants in removing SUC calls for the development and implementation of advanced technologies capable of effectively degrading this compound. Failure to address this issue could facilitate its continuous release and accumulation in aquatic ecosystems, with long-term ecological and public health implications.

Future research should therefore expand beyond short-term assessments and focus on chronic and multigenerational impacts of SUC exposure. Special attention should be given to its potential interactions with other pollutants, its role in cumulative ecological stress, and the evaluation of its transfer and biomagnification across trophic levels. Such knowledge will be crucial to support comprehensive risk assessments and the formulation of effective environmental policies aimed at mitigating the risks associated with this widely used artificial sweetener.

## Figures and Tables

**Table 1:** Sucralose Concentrations in Water Systems and Muscle Tissue of Common Carp at Different Exposure Times, Determined by HPLC.

Exposure	Exposure Time	SUC in Water System	SUC in Muscle Carp
Concentration	(h)	(μg L^−1^)	(μg g^−1^)
Control group	12	ND	ND
	24	ND	ND
	48	ND	ND
	72	ND	ND
	96	ND	ND
0.05 μg L^−1^	12	0.04 ± 0.008	0.0010 ± 0.0001
	24	0.04 ± 0.007	0.0010 ± 0.0001
	48	0.03 ± 0.002	0.0023 ± 0.0002
	72	0.03 ± 0.001	0.0042 ± 0.0003
	96	0.02 ± 0.001	0.0041 ± 0.0001
155 μg L^−1^	12	132.2 ± 3.1	6.2 ± 0.8
	24	127.5 ± 1.8	5.8 ± 1.1
	48	118.6 ± 2.1	7.6 ± 1.2
	72	112.7 ± 1.5	8.3 ± 2.3
	96	98.3 ± 2.9	8.1 ± 1.5

ND: non detectable.

**Table 2:** Results of texture profile analysis in muscle of *Cyprinus carpio* exposed to two sucralose concentrations (0.05 µg/L and 155 µg/L) for 12, 24, 48, 72 and 96 h.

		Exposure Time				
		12 h	24 h	48 h	72 h	96 h
Hardness	Control	1.22 ± 0.06 ^a^	1.22 ± 0.06 ^a^	1.22 ± 0.06 ^a^	1.22 ± 0.06 ^a^	1.22 ± 0.06 ^a^
	0.05 µg/L	1.15 ± 0.04 ^c^	1.13 ± 0.06 ^c^	1.11 ± 0.05 ^c^	1.19 ± 0.06 ^d^	1.33 ± 0.07 ^a^
	155 µg/L	1.22 ± 0.04 ^c^	1.19 ± 0.05 ^c^	1.10 ± 0.06 ^d^	1.44 ± 0.07 ^b^	1.49 ± 0.09 ^a^
Cohesiveness	Control	0.11 ± 0.06 ^a^	0.11 ± 0.06 ^a^	0.11 ± 0.06 ^a^	0.11 ± 0.06 ^a^	0.11 ± 0.06 ^a^
	0.05 µg/L	0.33 ± 0.01 ^c^	0.32 ± 0.02 ^c^	0.31 ± 0.01 ^c^	0.35 ± 0.03 ^b^	0.37 ± 0.02 ^a^
	155 µg/L	0.34 ± 0.06 ^c^	0.36 ± 0.06 ^b^	0.37 ± 0.06 ^a^	0.37 ± 0.06 ^a^	0.36 ± 0.06 ^b^
Elasticity	Control	0.46 ± 0.04 ^a^	0.46 ± 0.03 ^a^	0.46 ± 0.05 ^a^	0.46 ± 0.06 ^a^	0.46 ± 0.04 ^a^
	0.05 µg/L	0.42 ± 0.05 ^c^	0.41 ± 0.03 ^c^	0.39 ± 0.04 ^d^	0.47 ± 0.05 ^b^	0.51 ± 0.06 ^a^
	155 µg/L	0.44 ± 0.06 ^c^	0.42 ± 0.06 ^d^	0.40 ± 0.06 ^e^	0.46 ± 0.06 ^b^	0.49 ± 0.06 ^a^
Chewiness	Control	0.06 ± 0.06 ^a^	0.06 ± 0.06 ^a^	0.06 ± 0.06 ^a^	0.06 ± 0.06 ^a^	0.06 ± 0.06 ^a^
	0.05 µg/L	0.16 ± 0.06 ^c^	0.15 ± 0.06 ^c^	0.14 ± 0.06 ^c^	0.20 ± 0.06 ^b^	0.25 ± 0.06 ^a^
	155 µg/L	0.18 ± 0.06 ^b^	0.18 ± 0.06 ^b^	0.16 ± 0.06 ^b^	0.25 ± 0.06 ^a^	0.25 ± 0.06 ^a^
Gumminess	Control	0.13 ± 0.06 ^a^	0.13 ± 0.06 ^a^	0.13 ± 0.06 ^a^	0.13 ± 0.06 ^a^	0.13 ± 0.06 ^a^
	0.05 µg/L	0.38 ± 0.06 ^c^	0.36 ± 0.06 ^c^	0.35 ± 0.06 ^c^	0.42 ± 0.06 ^b^	0.49 ± 0.06 ^a^
	155 µg/L	0.42 ± 0.06 ^b^	0.43 ± 0.06 ^b^	0.40 ± 0.06 ^b^	0.55 ± 0.06 ^a^	0.52 ± 0.06 ^a^

Different letters indicate significant differences among treatments (*p* < 0.05).

## Data Availability

The original contributions presented in this study are included in the article. Further inquiries can be directed to the corresponding author.

## Abbreviations

The following abbreviations are used in this manuscript:

Ace-K	Acesulfame-K
CYC	Cyclamate
LPX	Lipid peroxidation
MDA	Malondialdehyde
MEC	Molar extinction coefficient
MP	Myofibrillar protein
PCC	Protein carbonyl content
SAC	Saccharin
SUC	Sucralose
SH	Sulfhydryl
TPA	Texture profile analysis
WHC	Water holding capacity.
